# An Improved Sea Lion Optimization for Workload Elasticity Prediction with Neural Networks

**DOI:** 10.1007/s44196-022-00156-8

**Published:** 2022-10-29

**Authors:** Binh Minh Nguyen, Trung Tran, Thieu Nguyen, Giang Nguyen

**Affiliations:** 1 School of Information and Communication Technology, Hanoi University of Science and Technology, Hanoi, Vietnam; 2 Institute of Informatics, Slovak Academy of Sciences, 84507 Bratislava, Slovakia; 3 Faculty of Informatics and Information Technologies, Slovak University of Technology, 84216 Bratislava, Slovakia

**Keywords:** Nature-inspired computing, Improved sea lion optimization, Memorizing historical movement, Levy flight trajectory, Opposition-based learning, Extreme learning machine, Neural network, Workload prediction

## Abstract

The work in this paper presents a study into nature-inspired optimization applied to workload elasticity prediction using neural networks. Currently, the trend is for proactive decision support in increasing or decreasing the available resource in cloud computing. The aim is to avoid overprovision leading to resource waste and to avoid resource under-provisioning. The combination of optimization and neural networks has potential for the performance, accuracy, and stability of the prediction solution. In this context, we initially proposed an improved variant of sea lion optimization (ISLO) to boost the efficiency of the original in solving optimization problems. The designed optimization results are validated against eight well-known metaheuristic algorithms on 20 benchmark functions of CEC’2014 and CEC’2015. After that, improved sea lion optimization (ISLO) is used to train a hybrid neural network. Finally, the trained neural model is used for resource auto-scaling based on workload prediction with 4 real and public datasets. The experiments show that our neural network model provides improved results in comparison with other models, especially in comparison with neural networks trained using the original sea lion optimization. The proposed ISLO proved efficiency and improvement in solving problems ranging from global optimization with swarm intelligence to the prediction of workload elasticity.

## Introduction

Cloud computing is already a mainstream model for resource-intensive applications. Most Infrastructure-as-a-Service (IaaS) offer at least one resource monitoring solution for customers, who can rely on collected data and thresholds to decide the amount of resources and scaling moments themselves. However, wasting and lacking resources problems occur because it is difficult to determine exactly the scaling moments using the threshold approach. To improve the quality of resource provision service, proactive workload prediction is investigated for effective resource management in advance [1].

Neural network have been developed and widely applied to classification, pattern recognition, and forecasting solutions. These networks are not intended to be realistic models of the brain, but rather robust algorithms and data structures capable of modeling difficult problems. Neural networks have units (neurons) organized in layers. They can be divided into shallow (one hidden layer) and deep (more hidden layers) networks. Through proper training, the network can learn how to optimally represent inputs to output variables, and therefore, learn how to make predictions.

With the current boom of bio- and nature-inspired methods, they are furthermore improved in various ways, such as stochastic components, hybridization, and evolution. The aim is to avoid local optima in the global optimization search process in a better way to archive faster computation time, training with less data while maintaining acceptable high accuracy without significantly increasing model complexity [2].

In this context, our contributions presented in this study are as follows.

- To study various effects such as memorizing historical movement (MHM), Levy flight trajector (LFT), and opposition-based learning (OBL) on performance improvement of metaheuristic optimization.
- To improve the original sea lion optimization (SLO) [3] by the combination of MHM and LFT in the exploration phase and OBL in the exploitation phase. The novel improved optimizer is called ISLO.
- To carefully evaluate the proposed optimizer with the CEC 2014 [4] and CEC 2015 functions [5] and to demonstrate its effectiveness.
- To use ISLO to train neural networks models to predict system workloads with real public datasets. The results showed that the model offers improvements in the convergence, stability, and prediction accuracy performance compared to other optimizers while integrating with neural networks for modeling.

The remainder of the paper is organized as follows. Section 2 provides an overview and the current situation with nature-inspired optimization, neural network modeling, including the training process and data processing, and preparation for time series modeling. Section 2.4 provides a detailed look at the design and implementation of the original sea lion optimization algorithm (SLO). Section 2.5 describes the work steps of the proposed solution. Section 3 describes our proposed improved variant of sea lion optimization ISLO in Sect. 3.1 and our hybrid neural network model, specifically the extreme learning machine (ELM) in Sect. 3.2 trained by ISLO (Sect. 3.3). Experiments including their setting, evaluation metrics, benchmark functions, and datasets are described in detail in Sect. 4. Experiment results and discussion of the results are presented in Sect. 5 with comparison with other neural network models trained by other approaches. Finally, the conclusions and future work are given in Sect. 6.

## Related Work

### Workload Elasticity Prediction

Prediction of workload elasticity is one of the application problems in cloud computing. It comes from the recording of data logs in cloud data centers [6] to provide better decision support for resource elasticity. Similar time-ordered data are available in other business sectors, such as weather prediction, financial stocks, or healthcare monitoring. Workload elasticity is also called resource auto-scaling, which is a big issue to be tackled to give cloud servers a flexible ability like being adaptive and scalable with automatically recovering and effective resource allocation.

There are many approaches to dealing with time-series data. The most mentioned methods come from statistics [7] such as autoregressive integrated-moving average (ARIMA), autoregressive moving average (ARMA), moving average (MA) and its variance like general autoregressive conditional heteroscedastic [8]. The next direction goes through machine learning and deep learning as reported in [9, 10] for larger datasets. Deep learning models are favorite with competitive performance. However, they require a larger amount of data to train and computational power.

### Neural Networks and Learning Ability

In the last three-decade, neural networks have been widely applied to real-world applications such as classification, pattern recognition regression, and forecasting problems [11, 12]. The most well-known and often-used model in this category is multi-layer perceptron (MLP) and its subcategories. These neural networks provide learning ability with simple structures. There are several ways to increase neural network performance:

1. Using more complex structured layers such as deep learning,
2. Replacing gradient descent training algorithm with nature-inspired algorithms,
3. Replacing hidden layers with different techniques to form different and more effective variants.

In the first way, deep learning requires more data for model training, which comes with requirements on computational power with the promise in predictive quality [13]. The second way tries to improve neural network performance is to use other methods to train neural networks instead of traditional gradient descent ones [14–16].

In the third way, here are attempts to increase neural network performance by modifying their structure without increasing the complexity (shallow learning). Functional-linked neural network is one such variant [17]. Instead of using the hidden layer to learn a non-linear relationship between input and output, they use a set of expansion functions to learn a non-linear relationship [18]. However, functional-linked neural network are domain-specific dependent, i.e., the correct expansion function has to be set based on concrete datasets to archive the best results.

Other types of MLP are feed-forward neural network, cascade forward neural network (CFNN) [19], ELM [20]. The difference between ELM and MLP is the calculation of the weights of the network. In ELM, the weights between input and hidden layer are randomly chosen. The weights between the hidden and output layer are calculated based on the generalized inverse operation of the hidden layer output matrix. ELM not only learns much faster with better generalization performance than traditional gradient-based learning algorithms but also avoids many difficulties faced by gradient-based learning methods such as stopping criteria, learning rate, learning epochs, and local minima. However, the problem of ELM is the requirement of more hidden neurons than traditional gradient-based learning algorithms and leads to the ill-condition problem due to randomly selecting input weights and hidden biases. In [21], the authors proposed an evolutionary ELM using the differential evolution (DE) algorithm to select input weights and using Moore–Penrose generalized inverse to analytically determine output weights. These improvements can bring good performance and make a compact ELM network.

In this work, the motivation is to propose a new low-cost hybrid model using nature-inspired computation to train neural network models for the prediction of workload elasticity with data logs from the underlying monitoring system.

### Nature-Inspired Computing

Recently, an impressive variety of nature-inspired algorithms (metaheuristic) has been investigated and reported [22, 23]. The optimization problems that attracted the attention of these approaches have a large variance, ranging from single-objective to multi-objective, continuous to discrete, constrained to unconstrained. Solving these problems is not a straightforward task due to their complex behavior [24, 25]. Nature-inspired algorithms provide a solution to many application problems [26, 27]. They are designed to achieve approximately optimal solutions in an acceptable time range for NP-hard (NP-hardness or non-deterministic polynomial-time hardness) problems [28].

Most of the classical metaheuristic algorithms have been developed a long time ago, like genetic algorithm (GA) [29, 30], particle swarm optimization (PSO) [31]. Despite their achievements, novel and improved evolutionary approaches have emerged successfully with a great number of new metaheuristics inspired by evolutionary or behavioral processes. These new-generation algorithms are often called nature-inspired algorithms. The entire group of these algorithms can be classified into four categories [32, 33].

- Evolutionary algorithms with the GA mentioned above, which mathematically mimics Darwinian evolution laws [34]. Differential evolution also belongs to this group with its adaptive variants. The search process starts with randomly generated solutions that evolve continuously throughout generations [35].
- Swarm-based algorithms or swarm intelligence refer to the collective behaviors of wild animals, e.g., birds, cats, and bacteria and mimic their social interactions [36]. The optimization process in these algorithms is mainly characterized by the ability to explore based on the diversity of platforms and develop exploitation based on searching for the best solution [37]. The typical examples are particle swarm optimization, whale optimization algorithm (WOA) [38], coyote optimization algorithm (COA) [39] artificial bee colony (ABC) [40], and hunger game search (HGS) [41].
- Physics-inspired algorithms mainly simulate physical phenomena that occur in nature by mathematical formulas, e.g., quantum-based sine cosine algorithm [42] or imitating physical principles in the universe such as galactic swarm optimization [43], multi-verse optimization [44], parallel hurricane optimization algorithm [45], movable damped wave algorithm [46], improved atom search optimization [47].
- Human-inspired algorithms are unique because they draw inspiration from several phenomena commonly associated with human behavior, lifestyle, or perception. Recent examples are coronavirus herd immunity optimization (CHIO) [48] qeuing search optimization (QSO) [33].

Among these types of nature-inspired algorithms, swarm intelligence is the most popular because it is easy to understand and implement. There are a number of techniques to improve their performance such as levy-flight trajectory [49], memory-based method [50], crossover operations [51], opposition-based learning [16] and hybridization [2].

In machine learning, nature-inspired algorithms are often used for feature selection and hyper-parameter tuning. In this work, the use of such algorithms is investigated to optimize neural networks [52], that is, using ISLO to train neural networks for workload prediction based on time-series data.

### Sea Lion Optimization (SLO)

SLO was introduced to solve global-scale optimization. It mimics the hunting behaviors of sea lions consisting of the way they encircle and capture prey or how they use their tail and whiskers. SLO can provide very competitive results compared with other well-known particle swarm optimization algorithms when working on different benchmark functions. More details about SLO are provided in the original work [3].

In this Section, the most important operations of SLO are summarized and the SLO pseudo-code is presented in Algorithm 1.
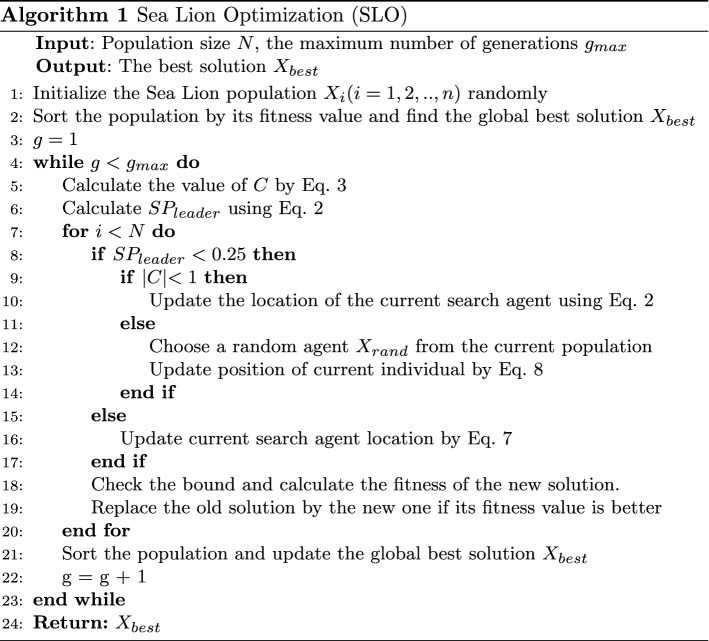

1. **Detecting and tracking phase**
  At first, SLO constructs *N* (the size of the population) *D*-dimensional solutions (Eq. 1) by using uniform random distribution in the search space as follows. Then, in the swarm of sea lions, they identify the location of the prey and gather other members who will join the subgroup to organize the net following the encircling mechanism. The prey is considered the best current solution or the solution closest to the optimal solution. These behaviors are presented in Eq. 2.
  1 $$\begin{aligned} X_{i, j}^{init} = X_{i, j}^{min} + rand_{i, j}~(X_{i, j}^{max} - X_{i, j}^{min}) \end{aligned}$$
  where:
  $$i=1,2, \cdots , N$$
  $$j=1,2, \cdots , D$$
  $X_{i, j}^{init}$ is the initial position vector of $i{th}$ solution;
  $X_{i, j}^{min}$ denote the minimum value for the $j{th}$ dimension of $i{th}$ solution;
  $X_{i, j}^{max}$ denote the maximum values for the $j{th}$ dimension of $i{th}$ solution;
  *rand* is a uniform random value in the interval [0, 1].
  Solutions are evaluated for their fitness using the objective function.
  2 $$\begin{aligned} X^{g+1}= \, & {} X_{best} - C~\mid 2~r~X_{best} - X^g \mid \end{aligned}$$
  3 $$\begin{aligned} C= \, & {} 2~\Bigg (1 - \frac{g}{g_{max}}\Bigg ) \end{aligned}$$
  where:
  $X_{best}^g$ is the position vector of the best solution;
  $X^g$ is the sea lion in iteration *g*;
  *g* is the current iteration of generations;
  $g_{max}$ is the maximum number of generations;
  *r* is a random value in the range [0, 1];
  that is multiplied by 2 to increase the search operation range;
  $X^{g+1}$ is the new position of the search agent after updating;
  *C* is a variable with linearly decreased values from 2 to 0 throughout the iteration, indicating the encircling mechanism of the sea lion group when they move towards the prey and surround them.
2. **Vocalization phase** When a sea lion recognizes a group of its prey (such as fish), it will call other sea lions in its group to gather and create a net to capture the prey. That sea lion is considered as *leader* and it will lead the group of sea lions toward and decide the behaviors of the group. These behaviors are mathematically modeled as shown in Eq. 4, Eq. 5 Eq. 6.
  4 $$\begin{aligned} SP_{leader}= & {} \mid (V_1(1+V_2)/V_2 \mid \end{aligned}$$
  5 $$\begin{aligned} V_1= & {} \sin (\theta ) \end{aligned}$$
  6 $$\begin{aligned} V_2= & {} \sin (\phi ) \end{aligned}$$
  where:
  $SP_{leader}$ is the value that illustrates the decision of *leader* followed by other sea lions in the group;
  $\theta$ is the angle of voice reflection in the water;
  $\phi$ is the angle of voice refraction in the water;
  In our work, $\theta = 2\pi r$ and $\phi = 2\pi (1-r)$ where *r* is a random number in the range [0, 1].
3. **Attacking phase (Exploitation phase)** The hunting activities of sea lions led by *leader* are described in two phases as follows:
  *Dwindling encircling technique:* This behavior depends on the value of *C* in Eq. 2. *C* is linearly decreased from 2 to 0 throughout the iteration, so this allows the search space around the current best position to shrink and force other search agents to update in this search space as well. Therefore, a newly updated position of a sea lion can be located anywhere in the search space between its current position and the location of the best agent. present.
  *Circling updating position*: Sea lions chase the bait ball of fishes and hunt them starting from the edges by Eq. 7, with *m* a random number in the range $[-1, 1]$.
    7 $$\begin{aligned} X^{g+1} = X_{best} + \cos (2 \pi m)~\mid X_{best} - X^g \mid \end{aligned}$$
4. **Searching for prey (Exploration phase)** In the exploration phase, the search agents update their positions based on a randomly selected sea lion. The condition that allows the exploitation phase to take place is when the value of *C* becomes greater than 1, and the process of finding a new agent is presented by Eq. 8.
  8 $$\begin{aligned} X^{g+1} = X_{rand}^g - C~\mid 2~r~X_{rand}^g - X^g \mid \end{aligned}$$
  where $X_{rand}^t$ is a random sea lion randomly selected from the current population. *r* is a random value in the range [0, 1].

The results of the work [3] show that SLO faces obvious problems with nature-inspired algorithms, such as being trapped in local optima and slow convergence. In this work, both exploitation and exploration phase for ISLO (Section 3) is improved compared to the original SLO.

### The Work Steps of the Proposed Solution

Based on the context presented above, the remainder of this work proceeds through the following steps.

1. To propose an improved variant of SLO called Improved Sea Lion Optimization (ISLO) by embracing the idea of MHM of sea lions into account to upgrade the exploration ability in combination with LFT and the idea of OBL to enhance SLO exploitation capacity.
2. To test the convergence ability of ISLO by benchmark functions of 4 function types: unimodal, multimodal, hybrid, and composition functions. After that, ISLO performance is compared with the original SLO and six well-known optimization algorithms:
  Genetic algorithm and an improved version of the DE algorithm - surrogate assisted parameter adapted DE (SAP-DE) [53] in the evolutionary-based group;
  COA algorithm, HGS algorithm and a modified version of WOA - hybrid improved WOA (HI-WOA) [54] in the swarm-based group;
  CHIO and a modified version of life choice-based optimization (LCBO) called modified version of LCBO (M-LCO) [55] in the human-based group.
  The results show that ISLO provides superior final fitness values and decent convergence speed compared to the others.
3. To propose a hybrid model called ISLO-ELM, in which ISLO is used for training ELM. The aim is to model workload elasticity prediction based time-series data logs for auto-scaling demand in cloud data centers without significantly increasing complexity.
4. ISLO-ELM performance is validated on 4 real and public datasets of resource workload of server clusters and Internet traffic. The results are compared with MLP, CFNN, FLNN and ELM in terms of forecast quality. The optimization capacity of ISLO is also tested against enhanced enhanced tug of war optimization (OTWO) [56] and SLO to optimize ELM (OTWO-ELM and SLO-ELM). The outcome shows that the model is very competitive and has better potential results compared to the others.

## ISLO and Workload Elasticity Prediction

### Improved Sea Lion Optimization (ISLO)

**Exploration phase improvement**

In the SLO exploration phase, newborn agents cause poor exploration search ability due to the inheriting features of existing solutions (randomly chosen agent $X_{rand}^g$ but still in the current population). To tackle this problem, a newly created solution needs to satisfy two requirements: 1) carrying random features to ensure a strong capability of the exploration phase, and 2) landing in a position decent enough (close enough to the best agent position).

Based on that motivation and to enhance the performance of Eq. 8, the advantage of both the best global solution and the individual’s history is taken in a new improved operation. The idea of an individual’s historical information originally comes from PSO, which is widely used in many algorithms such as gaining-sharing knowledge algorithm [57] and bird swarm algorithm [58]. A piece of information from global best information ensures the second requirement; meanwhile, the information of individual’s historical with random coefficient ensures the first requirement for the newly updated solution. When combining three vectors, newly generated solutions will be able not only to explore the search space but also to explore the best global solution and the best individual’s experiments. Following that direction, the new update mechanism in SLO by Eq. 9, Eq. 10 and Eq. 11 is proposed to improve the exploitation ability as follows:

9 $$\begin{aligned} dif_1= \, & {} (2~r_1~X_{best}^g - X^g) \end{aligned}$$

10 $$\begin{aligned} dif_2= \, & {} (2~r_2~X_{local}^g - X^g) \end{aligned}$$

11 $$\begin{aligned} X^{g+1}= \, & {} X^g + C \cdot dif_1 + C \cdot dif_2 \end{aligned}$$

where:

- $X_{local}^g$ is the personal best position up to the iteration *g*;
- *r*1, *r*2 are random numbers in the range [0, 1];
- $dif_1$ the difference between the current position and the best solution found so far;
- $dif_2$ the difference between the current position and the best solution found in the history of the current individual.

Especially with the parameter *C*, in a few iterations, Eq. 11 focuses on the exploration process with larger information from both vectors, helping the algorithm to find the most promising area in a larger jump. In later iterations, the algorithm explores with the smaller jump from both vectors.

In the new Eq. 11, the newly updated position of an individual is the result of adding two vectors to the original agent, one is the vector that presents the direction of that individual towards the best agent, and another is the direction towards its own experiences in history. The influences of both two factors are determined by two random numbers $r_1$ and $r_2$. They also play an extremely important role in the update mechanism because they create random characteristics for the operation, helping ISLO avoid the local minimum and taking advantage of the two factors. Without the appearance of $r_1$ and $r_2$, the updated position is always affected by the same portion of the best agent and the same portion of its experience over generations, which may lead to the degradation of the diversity of the population.

**Exploitation phase improvement**

From our observations, SLO takes advantage of the global best solution and moving around to create new exploited solutions. However, their operation (Eq. 2) is based on the minus sign and absolute function. It makes the newly updated solution always toward one direction of the global best solution. Therefore, limiting the exploitation ability of the algorithm in multi-dimensional space, where the true global best solution may hide in the other direction of the current global best solution. To address this problem, the minus sign and absolute function in Eq. 2 is removed. The OBL process helps ISLO to search faster in exploitation [56]. OBL has successfully applied for grasshopper optimization algorithm [59], grey wolf optimization [60], etc.

**Fig. 1:**
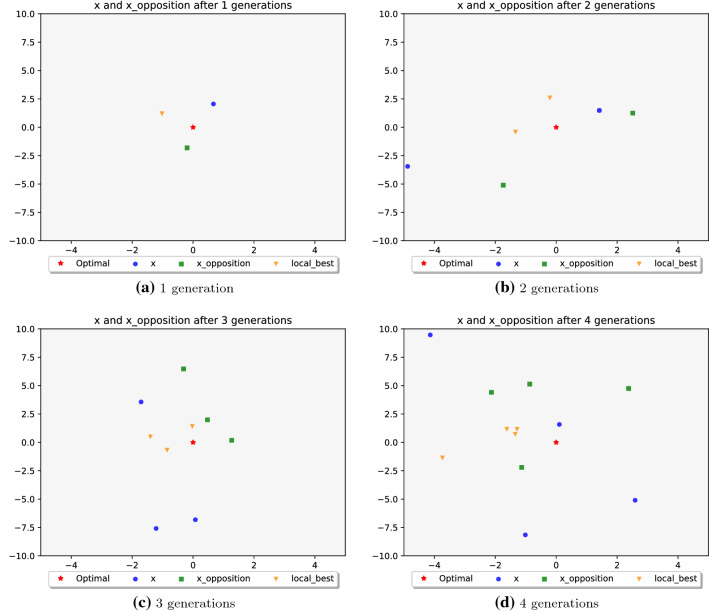
Position visualization of $X^{g+1}$ and $X^{g+1}_{oppo}$ after 1 (**a**), 2 (**b**), 3 (**c**), 4 (**d**) generations on 2-D scale

At first, Eq. 12 improves the exploitation. The *N*(0, 1) is a normal variable to ensure that the newly created solution is exploited in a random direction for each generation, and also to ensure that the created solution jumps in a small range near the $X_{best}$ solution due to the large value of *C* (linear decrease from 2 to 0).

After that, OBL is applied to create an opposite solution (Eq. 13) of the above generated solution. Consequently, ISLO searches for both the current position and its opposite position via the global best solution simultaneously, helping ISLO to exploit faster and better. Figure 1 visualizes the position of $X^{g+1}$ and $X^{g+1}_{oppo}$ after 1, 2, 3, 4 generations on the 2-D scale. In which the orange triangle (local best) is the $X_{best}$ found so far, the red star is the global optimal point.

1. Create a new solution using
  12 $$\begin{aligned} X^{g+1} = X_{best} + C~N(0, 1)~(2~r_3~X_{best} - X^{g}) \end{aligned}$$
2. Create an opposite solution $X_{oppo}^{g+1}$ by calculating the opposing position to $X^{g+1}$ through $X_{best}$.
  13 $$\begin{aligned} X_{oppo}^{g+1} = LB + UB - X_{best} + r_4~(X_{best} - X^{g+1}) \end{aligned}$$

**Additional improvement**

In SLO, the circling updating process presents the chasing bait ball of fishes and hunt them starting from the edges. The position of the sea lion is changed from the current position toward a nearby position of the global best solution by using the coefficient of the cosine function (Eq. 7). This may not be enough to help sea lions catch the biggest ball of fish because cosine is a periodic function. After being chased by different sea lions at the same time, fishes change their direction leading to the different movements of the bait ball. Therefore, a more complicated trajectory of sea lions helps them to catch more fish. Based on that motivation, in the circling phase of sea lions, an additional operation using the Levy-flight trajectory (LFT) is proposed for ISLO.

Levy flight [61] is a probability distribution proposed to simulate bird foraging routes. As a global search operator, LFT searches for space using short-distance walking combined with long-distance jumping routes. These two abilities help to improve the diversity and local exploitation ability of the population, especially with the approximate formula proposed by Mantegna [62]. In general, the Levy step size can be expressed as:

14 $$\begin{aligned}&Levy(s) \sim |s|^{-1-\beta } \qquad with \qquad 0 < \beta \le 2 \end{aligned}$$

15 $$\begin{aligned}&s = \frac{\mu }{|v|^{1/\beta }} \end{aligned}$$

16 $$\begin{aligned}&\mu \sim N(0, \sigma _{\mu }^2) \end{aligned}$$

17 $$\begin{aligned}&v \sim N(0, \sigma _{v}^2) \end{aligned}$$

18 $$\begin{aligned}&\sigma _{\mu } = \Bigg [ \frac{\Gamma (1 + \beta ).\sin (\pi .\beta /2)}{\Gamma ((1 + \beta )/2).\beta .2^{(\beta - 1)/2}} \Bigg ] ^{1/\beta } \end{aligned}$$

19 $$\begin{aligned}&\sigma _{v} = 1 \end{aligned}$$

where:

- *s* is the step length of the LFT calculated by Mantegna algorithm,
- $\mu$, *v* are chosen from normal distribution,
- $$\beta$$ in range (0, 2],
- $\Gamma$ is a gamma function.

The purpose of using the Levy-flight technique for SLO is to enhance the diversity and the local exploitation ability to find global optima by its complex trajectory. So, the proposed Levy-flight updating equation is as follows.

20 $$\begin{aligned} X^{g+1} = X_{best} + ss~Levy(S) \otimes (X_{best} - X^g) \end{aligned}$$

where:

- *ss* is the step size related to the scales of the problem,
- used to avoid Levy-flight jumping out of the search space
- (for our use case, $ss = 0.001$)
- $\otimes$ is entry-wise multiplications,
- *Levy*(*S*) is a set of Levy step lengths in a D-dimensional space.

ISLO improves both the exploitation and exploration phases of SLO by taking into account the combination of the MHM of individuals and LFT for the exploration phase and using the OBL operation and LFT for the exploitation phase. These improvements (MHM, LFT, and OBL) form ISLO as the improved SLO optimizer. The ISLO pseudo-code is presented in Algorithm 2.
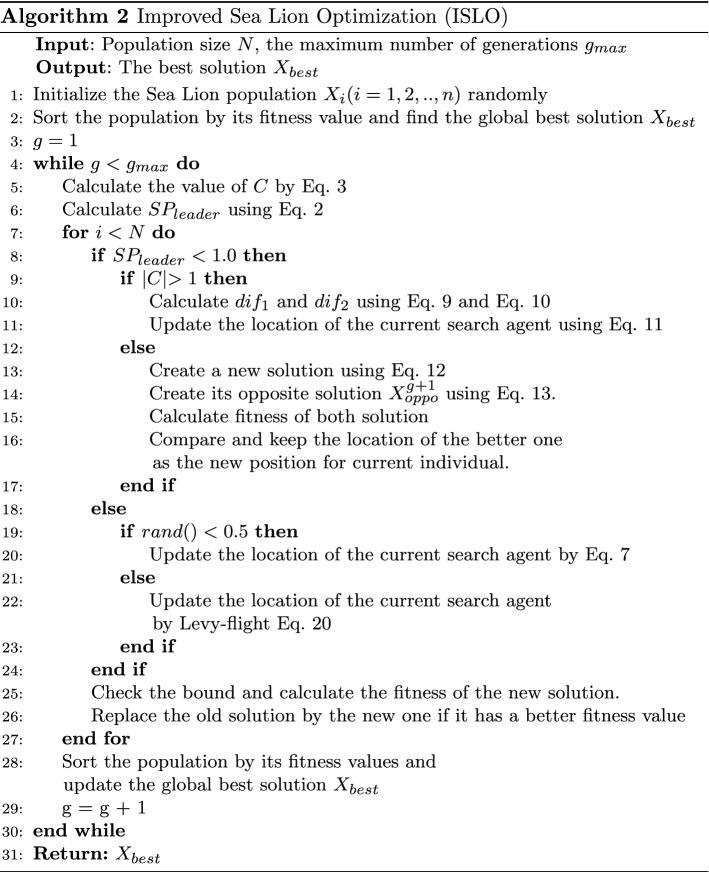


### Extreme Learning Machine (ELM)

**Fig. 2:**
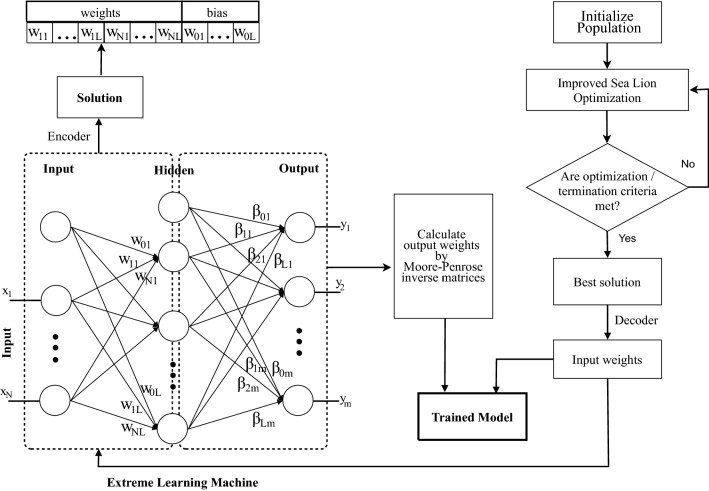
ELM architecture and encoding process

The difference between ELM and MLP is the training algorithm. ELM does not require gradient-based back propagation to work but uses a random process and Moore-Penrose generalized inverse to set its weights. The architecture of the single-hidden-layer ELM illustrated in Fig. 2—the left side, and the mathematical formulas for the ELM model are presented below. The ELM output is calculated as follows:

21 $$\begin{aligned} f(x) = \sum ^L_{i=1} \beta _i~g_i(x) = \sum ^L_{i=1} \beta _i~g(w_i~x_j + b_i) \qquad j=1,...,N \end{aligned}$$

where:

- *L* is the number of hidden units,
- *N* is the number of training samples,
- *g* is the activation function,
- *x* is an input vector,
- *w* is the weight vector between the input and hidden layer,
- *b* is the bias vector between the input and hidden layer,
- $\beta$ is the weight vector between the hidden and output layer
- (called the hidden weight that includes both weights and biases).
- This $\beta$ is a special matrix calculated by a pseudo-inverse operation.

The shortening of the matrix equation can be written as follows.

22 $$\begin{aligned} T= \, & {} H \beta \end{aligned}$$

23 $$\begin{aligned} M= & {} \begin{bmatrix} g(w_1*x_1 + b_1) &{} . &{} . &{} g(w_L*x1 + b_L)\\ . &{} . &{} &{} \\ . &{} &{}. &{} \\ g(w_1*x_N + b_1) &{} &{} &{} g(w_L*x_N + b_L) \end{bmatrix}_{N, L} \end{aligned}$$

24 $$\begin{aligned} \beta= & {} \begin{bmatrix} \beta _1^T \\ . \\ . \\ \beta _L^T \end{bmatrix}_{L, m} \end{aligned}$$

25 $$\begin{aligned} T= & {} \begin{bmatrix} t_1^T \\ . \\ . \\ t_L^T \end{bmatrix}_{N, m} \end{aligned}$$

where:

- *m* is the number of outputs;
- *H* is called hidden layer output matrix;
- *T* is the training data target matrix.

Then the optimization objective is calculated as

26 $$\begin{aligned} ||H\hat{\beta } - T|| = \mathop {min}_{\beta } ||H \beta - T|| \end{aligned}$$

Because *H* is invertible, $\hat{\beta }$ can be calculated as

27 $$\begin{aligned} \hat{\beta } = H^+T \end{aligned}$$

After having $\hat{\beta }$, we can make a prediction on the new data. Finally, the ELM training process has the following steps:

1. Randomly assign weight $w_i$ and bias $b_i$, $i = 1,...,L$
2. Calculate hidden layer output *H*
3. Calculate output weight matrix $\hat{\beta } = H^+T$
4. Use $\hat{\beta }$ to make a prediction on new data $T' = H\hat{\beta }$

### Training ELM Model by ISLO

**Fig. 3:**
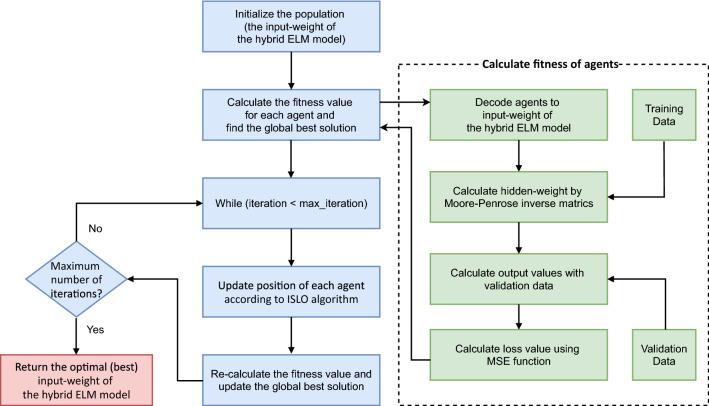
The work flow of ISLO-ELM hybrid model

The strength of ELM is speed, because it requires a little time to learn the relation between input and output by the random process and then just calculates the Moore-Penrose inverse matrix. This is a trade-off between speed and generalization performance. Non-optimal input weights may be randomly chosen, and this causes bad performance. In order to tackle the problem, in this paper, ISLO is used to replace the random process to find the optimal input-weight for the ELM network (Fig. 2). This way forms the ISLO-ELM model. There are two key aspects needed to be taken into consideration, which are the formation of an agent in ISLO, and the selection of fitness function.

1. *Agent formulation:* each agent in the population in ISLO is presented as one solution for the hybrid ELM model, which means that a search agent is a one-dimensional vector created by concatenating all weights and biases between the input and hidden layer. Therefore, the length of a solution can be calculated by Eq. 28.
  28 $$\begin{aligned} size(solution) = (1+n_i)~n_h \end{aligned}$$
  where $n_i, n_h$ is the number of input and hidden neurons, respectively.
2. *Fitness function:* fitness value of each agent in ISLO is considered as the loss value of the ELM model with the set of parameters of the agent and the input data. The loss function Mean Square Error (MSE) is used to calculate the difference between the actual and predicted output values by the generated agent for all samples in the training set.

The ISLO workflow applied in this work to train ELM is depicted in Fig. 3, and can generally be presented by the following steps:

1. *Initialization:* pre-defined the number of search agents in ISLO. Each set of input weights of the hybrid ELM model is encoded to a vector that plays a role as an agent in the ISLO population. (Fig. 2)
2. *Calculate fitness value for each search agent:* A solution is decoded into the input weight of the network. Calculate the hidden weight of the network by Moore-Penrose inverse matrices based on training data. Data samples in the validation set are then feed-forwarded through the network, generating predicted output values. Finally, the fitness value is calculated as the difference between the predicted output and the ground truth value using the MSE loss function.
3. Find the global best solution based on fitness value
4. Loop through maximum number of iterations
5. Update position of each agent by ISLO formulas
6. Re-calculate the fitness value and update the global best solution
7. *Repeat step 4 and step 6* until the difference is small (close) enough or the maximum number of generations is reached.
8. *Return the best input-weight set of ELM model.*

## Experiments

The ISLO optimization capability is tested by two folds: with benchmark functions (theoretical experiments) and with real datasets (practical experiments).

- For the theoretical experiments, 20 benchmark functions are used. This set of benchmark functions covers a wide range of functional groups, including classical unimodal and multimodal functions, hybrid functions, and composition functions taken from the special session of CEC 2014 and CEC 2015 [4, 5]. ISLO is compared with other algorithms in all four groups of meta-heuristic optimization include evolutionary, swarm-based, physical-based, and human-based algorithms.
- For practical experiments, the ISLO-ELM hybrid model is proposed, where ISLO is used to optimize ELM. Different real public datasets are used: the Google trace dataset (CPU and RAM), Internet traffic from the UK, and EU countries. ISLO-ELM is compared with classic models such as MLP, FLNN, CFNN, and ELM. It was also compared with other hybrid models (OTWO-ELM and SLO-ELM) to demonstrate the capability of ISLO in optimization.

### Theoretical Experiments

#### Benchmark Functions

The performance of ISLO has theoretically experimented with 20 benchmark functions in 4 groups:

- Unimodal functions that have only one global optimal point in the search space.
- Multimodal functions that have one global optimal point along with several local minimums.
- Hybrid functions: variables are randomly divided into some sub-components and then different basic unimodal and multimodal functions are used for different sub-components.
- Composition functions, which merge the properties of the sub-functions better and maintains continuity around the global/local optima.

**Fig. 4:**
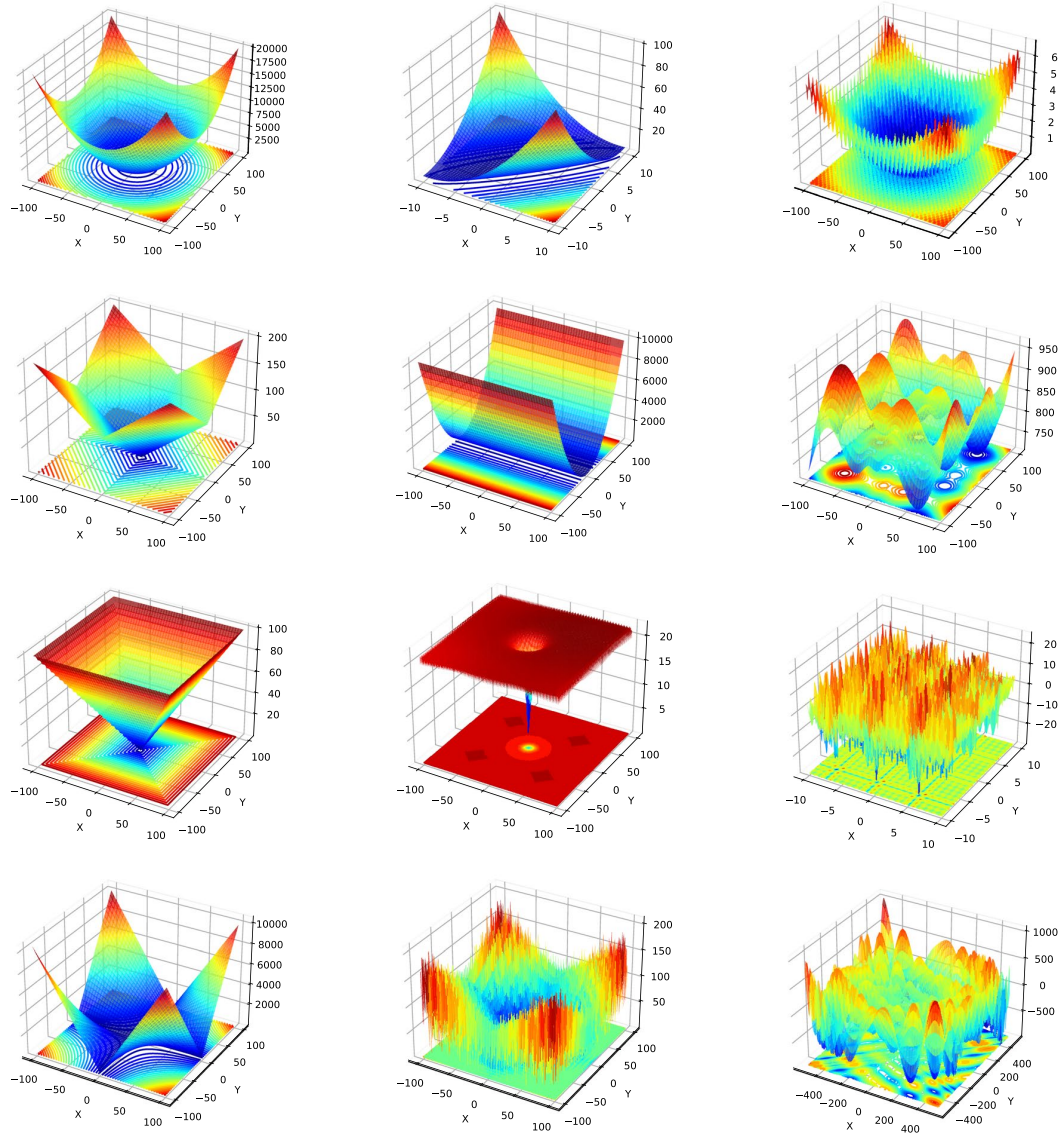
Examples of 3D plot for some of benchmark functions

A brief introduction about the function name, formula, search space, and optimal value of each function is shown in Table 1. More details about the formula and characteristics are in [4] and [5]. The 3D plots of several benchmark functions are presented in Fig. 4.

**Table 1:** Description of benchmark functions

Type	Mathematical definition	Range	$f_{min}$
Unimodal	$f_1(x) = \sum _{i=1}^n i*x_i^2$	[− 100, 100]	0
	$f_2(x) = \sum _{i=1}^n x_i^2 + (\frac{1}{2}*\sum _{i=1}^n i*x_i)^2 + (\frac{1}{2}* \sum _{i=1}^n i*x_i)^4$	[− 100, 100]	0
	$f_3(x) = \sum _{i=1}^n |x_i| + \prod _{i=1}^n |x_i|$	[− 100, 100]	0
	$f_4(x) = (x_1-1)^2 + \sum _{i=2}^n i*(2x_i^2 - x_{i-1})^2$	[− 100, 100]	0
	$f_5(x) = \sum _{i=1}^{n-1} [100(x_{i+1} - x_i^2)^2 + (x_i-1)^2]$	[− 100, 100]	0
Multimodal	$f_6(x) = -a.exp(-b\sqrt{\frac{1}{n}\sum _{i=1}^{n}x_i^2}) + a + exp(1) - exp(\frac{1}{n}\sum _{i=1}^{n}cos(cx_i))$ with a = 20 and b = 0.2	[− 100, 100]	0
	$f_7(x) =\left[ \left( ||{\textbf {x}}||^2 - n\right) ^2\right] ^\alpha + \frac{1}{n}\left( \frac{1}{2}||{\textbf {x}}||^2+\sum _{i=1}^{n}x_i\right) +\frac{1}{2}$	[− 100, 100]	0
	$f_{8}(x) = 10D + \sum _{i=1}^n (x_i^2 - 10*cos(2\pi *x_i))$	[− 100, 100]	0
	$f_9(x) = \sum _{i=1}^{n}{\sum _{j=1}^5{j sin((j+1)x_i+j)}}$	[− 100, 100]	0
	$f_{10}(x)=1-cos(2\pi \sqrt{\sum _{i=1}^{D}x_i^2})+0.1\sqrt{\sum _{i=1}^{D}x_i^2}$	[− 100, 100]	0
Hybrid	$f_{11}$ (function 17 in CEC 2014)	[− 100, 100]	1700
	$f_{12}$ (function 18 in CEC 2014)	[− 100, 100]	1800
	$f_{13}$ (function 20 in CEC 2014)	[− 100, 100]	2000
	$f_{14}$ (function 6 in CEC 2014)	[− 100, 100]	600
	$f_{15}$ (function 8 in CEC 2014)	[− 100, 100]	800
Composition	$f_{16}$ (function 9 in CEC 2015)	[− 100, 100]	900
	$f_{17}$ (function 10 in CEC 2015)	[− 100, 100]	100
	$f_{18}$ (function 12 in CEC 2015)	[− 100, 100]	1200
	$f_{19}$ (function 14 in CEC 2015)	[− 100, 100]	1400
	$f_{20}$ (function 15 in CEC 2015)	[− 100, 100]	1500

#### Model Comparison

The ISLO results with 20 benchmark functions are compared with eight other algorithms. To be fair in the comparison experiment, all algorithms are set with the same number of search agents (population size $p_s$ = 50) and the same number of maximum generations ($g_{max}$ = 1000). The number of dimensions for each function is 30 dimensions. The specific parameter for each algorithm is selected on the basis of the original paper and combined with the trial-and-error method in advance. The optimal parameter for each algorithm can be found below:

- For GA [63], the crossover probability $p_c=0.9$ and the mutation probability $p_m=0.025$
- For SAP-DE, weighting factor $wf=0.8$, crossover probability $cr=0.9$, F factor $F=1.0$.
- For PSO [64], the cognitive learning rates $c_1=c_2=2.05$, and the inertia factor *w* are set linearly and reduce from 0.9 to 0.4 over the course of iteration.
- For HGS, the probability of updating position $L=0.08$ and the highest hunger $LH=10000$.
- For CHIO, the basic reproduction rate $brr=0.06$ and the maximum age of infected cases $max\_age = 150$.
- For SLO and ISLO, hyper-parameters are set as described in the original paper [3].

#### Measurement Methods and Parameters Settings

The experimental results of each model are produced by calculating the mean (Eq. 29) and standard deviation *std* (Eq. 30) of 50 times running with the algorithms and functions mentioned above.

29 $$\begin{aligned} mean= & {} \frac{1}{N}\sum _{i=1}^N x_i \end{aligned}$$

30 $$\begin{aligned} std= & {} \sqrt{\frac{1}{N}\sum _{i=1}^n(x_i - \mu )^2} \end{aligned}$$

where:

- $$i = 1, 2, ..., N$$
- *N* is the size of the observation population;
- $$r_i$$ are observations;
- $\mu$ is the population mean.

For each function, after calculating the values of *mean* and *std* of each algorithm, the best algorithm will be denoted by $1^{st}$ ranked and determined by the following rules:

1. The *mean* values are considered. If an algorithm has the best value *mean*, it will be ranked as the best optimizer ($1^{st}$ ranked).
2. In the case where two or more algorithms have the same *mean* value, the one that has the most stable *std* value will be chosen as the best.
3. The mean ranking for each type of benchmark function (unimodal, multimodal, hybrid and composition) is calculated to illustrate which algorithm performed best in each set of functions.
  For example, unimodal has 5 functions $f_1$ to $f_5$, then the mean ranking of the ISLO algorithm in the unimodal set is calculated as
  31 $$\begin{aligned} \Bigg( rank_{ISLO}^{f_1} + ... + rank_{ISLO}^{f_5} \Bigg) / 5 \end{aligned}$$

### Practical Experiments

For the practical problem, our proposed ISLO-ELM model is utilized to solve time series forecasting on the cloud computing platform. Four datasets are used including CPU and RAM from Google trace cluster, Internet traffic from the UK, and EU countries.

The results of the proposed model ISLO-ELM are compared with classic models such as MLP, CFNN, FLNN and the original ELM. The optimizing capability of ISLO on ELM is also validated against hybrids OTWO (OTWO-ELM) and SLO (SLO-ELM).

#### Datasets

- Google cluster trace dataset: The most important dataset in our experiments is gathered by Google on a cluster of about 12500 machines [65] for 29 days, starting from May 2011. Resources requirements and usage data for each job are recorded by each machine in the cluster, and then the data is managed by the cluster’s management system. In the Google Trace dataset, two extremely important columns contain information of the central processing unit (CPU) and random access memory (RAM) required for each job. For that reason, we decide to choose these two data types as two time-series datasets (called Google Trace CPU and Google Trace RAM from here). The datasets are processed and summarized in the 5-minute interval, containing 8351 data points, and considered as the total demand for resources in the whole Google cluster.
- Internet traffic from the EU and EU countries: These two sets of data, which are used for experiments in [66], are recorded by two different ISPs. The EU Internet Traffic dataset comes from a private ISP playing a role as a reporter with centers in 11 European cities. The data correspond to a transatlantic link and were collected from 06:57 hours on 7 June to 11:17 hours on 29 July 2005. The UK Internet Traffic represents aggregated traffic in the United Kingdom’s academic network backbone. It was reported between 19 November 2004, at 09:30 hours and 27 January 2005, at 11:11 hours. Both two datasets are processed and summarized every 5 minutes, creating EU Internet Traffic (14773 records) and UK Internet Traffic (19989 records) as the input in our experiments.

The feature engineering goes thought transformation raw logs into time-series data [67]. After that, missing values are checked. The smooth sliding transformation is an operation that helps to remove short-term variations in order to reveal long-term trends is done by seasonal-trend decomposition using locally estimated scatterplot smoothing (STL). The cleaned data are also checked against white noise, randomness and unit root with the augmented Dickey-Fuller (ADF) test.

The characteristics of time-series data are ordered time-dependency sequences. There is a temporal dependency between observations that must be preserved during testing and validation. The method used for cross-validating in this work is built on a rolling basis known as the Time-Series-Split approach in machine learning. In our experiments, the size of the sliding windows is not changed, i.e. rolling basis with fixed window’s size and fixed split ratio (70:15:15) for train, test, and validation data.

#### Parameter Setting and Evaluation Metrics

As mentioned above, ISLO-ELM’s performance is compared with five classic models: MLP, CFNN, FLNN, ELM, and three hybrid-ELM models: OTWO-ELM, SLO-ELM, ISLO-ELM. The hyper-parameter settings for each model are described below:

- MLP, CFNN, ELM, and hybrid-ELM settings with the same architectures include one input layer, one hidden layer, and one output layer. The input size for all models is based on the feature engineering for each dataset.
- FLNN with single input and output layer, the expansion function is selected by the trial method as mentioned in [6].
- The number of epoch in NN models is set to 1000. The maximum number of generations in metaheuristic algorithms is also set to 1000. This setting is sufficient for all algorithms to converge to their final results.

In the training phase of general network, mean squared error (MSE) is used as the loss function. In the testing phase, mean absolute error (MAE), root mean squared error (RMSE), mean absolute percentage error (MAPE), ackge, Kullback-Leibler divergence (KLD) [68] are used as measurements for comparison. The Kling-Gupta efficiency (KGE) [69] combines the three components of Nash-Sutcliffe efficiency (NSE) of model errors (i.e. correlation, bias, ratio of variances or coefficients of variation) in a more balanced way. It has value range from $-Inf$ to 1. Essentially, the closer to 1, the more accurate the model is. The KLD [70] is used to measure how much a given arbitrary distribution is away from the true distribution. If two distributions perfectly match, $KLD(P||Q) = 0$ otherwise it can take values between 0 and $\infty$. Lower the KLD value, the better matched the true distribution with our approximation. The mathematical form of these metrics are as follows.

32 $$\begin{aligned} MSE= \, & {} \frac{1}{n} \sum ^n_{i=1}(y_i - \hat{y}_i)^2 \end{aligned}$$

33 $$\begin{aligned} RMSE= & {} \sqrt{MSE} \end{aligned}$$

34 $$\begin{aligned} MAE= \, & {} \frac{1}{n} \sum ^n_{i=1} |y_i - \hat{y}_i | \end{aligned}$$

35 $$\begin{aligned} MAPE= \, & {} \frac{1}{n} \sum ^n_{i=1} \bigg |\frac{y_i - \hat{y}_i}{y_i} \bigg |\end{aligned}$$

36 $$\begin{aligned} KGE= & {} 1 - \sqrt{(r - 1)^2 + (\beta - 1)^2 + (\gamma - 1)^2} \end{aligned}$$

37 $$\begin{aligned} KLD(P || Q)= & {} - \sum _{x \in X} P(x)log Q(x) + \sum _{x \in X} P(x) log P(x) = H(P, Q) - H(P) \end{aligned}$$

where:

- $y_i$ are observed value,
- $\hat{y}_i$ are predicted value,
- *r* is correlation coefficient,
- $\beta$ $\beta = \frac{\mu _{\hat{y}}}{\mu _y}$ is bias ratio,
- $\gamma$ $\gamma = \frac{CV_{\hat{y}}}{CV_y} = \frac{\sigma _{\hat{y}} / \mu _{\hat{y}}}{\sigma _y / \mu _y}$ is variability ratio,
- *CV* is coefficient of variation,
- $$\mu$$ is mean,
- $\sigma$ is standard deviation,
- *H*(*P*, *Q*) is the cross entropy of *P* and *Q*
- *H*(*P*) is the entropy of *P*.

## Results and Discussion

### Benchmark Functions Results

#### Unimodal and Multimodal Functions Results

**Table 2:** Comparison of optimization results obtained for unimodal functions (f1–f5) and multimodal functions (f6–f10)

Function		GA	SAP-DE	HI-WOA	COA	HGS	M-LCO	CHIO	SLO	ISLO
f1	Mean	2.13E-08	1.21E-04	0.00E+00	1.51E-03	0.00E+00	0.00E+00	2.69E-02	0.00E+00	0.00E+00
	Std	3.53E-08	2.15E-04	0.00E+00	6.32E-03	0.00E+00	0.00E+00	3.59E-02	0.00E+00	0.00E+00
	Rank	6	7	3	8	3	3	9	3	3
f2	Mean	2.71E+03	2.56E+04	8.88E+04	8.60E+04	0.00E+00	0.00E+00	8.06E+04	2.60E+04	0.00E+00
	Std	4.22E+02	2.15E+04	1.70E+04	2.42E+04	0.00E+00	0.00E+00	1.37E+04	1.23E+04	0.00E+00
	Rank	4	5	9	8	2	2	7	6	2
f3	Mean	1.24E+13	3.75E+15	2.23E-127	1.94E+01	0.00E+00	0.00E+00	5.11E+19	8.73E-49	0.00E+00
	Std	2.67E+13	1.62E+16	6.11E-127	1.01E+01	0.00E+00	0.00E+00	1.46E+20	2.55E-48	0.00E+00
	Rank	7	8	4	6	2	2	9	5	2
f4	Mean	4.67E+06	3.59E+06	6.67E-01	4.37E+06	6.67E-01	9.80E-01	5.81E+09	6.70E-01	6.67E-01
	Std	1.41E+06	8.21E+06	7.14E-06	3.78E+06	1.43E-03	9.92E-03	2.14E+09	7.96E-03	5.42E-06
	Rank	8	6	1	7	3	5	9	4	2
f5	Mean	8.30E+06	1.71E+07	2.63E+01	2.62E+07	2.77E+01	2.89E+01	1.22E+10	2.85E+01	2.75E+01
	Std	2.81E+06	4.93E+07	8.71E-01	2.86E+07	2.45E-01	3.44E-02	3.79E+09	1.71E-01	4.07E-01
	Rank	6	7	1	8	3	5	9	4	2
f6	Mean	8.5E+00	1.0E+01	1.3E-15	2.0E+01	4.4E-16	1.1E+00	2.0E+01	1.8E+01	4.4E-16
	Std	5.2E-01	7.0E+00	2.0E-15	6.3E-04	0.0E+00	4.7E+00	6.5E-03	6.3E+00	0.0E+00
	Rank	5	6	3	8	1.5	4	9	7	1.5
f7	Mean	1.2E+00	1.3E+00	6.3E-04	1.0E+00	0.0E+00	0.0E+00	1.2E+01	3.2E-03	0.0E+00
	Std	2.7E-02	4.1E-01	2.8E-03	7.6E-02	0.0E+00	0.0E+00	8.5E-01	1.4E-02	0.0E+00
	Rank	7	8	4	6	2	2	9	5	2
f8	Mean	1.0E+03	1.5E+03	3.7E+01	6.9E+02	0.0E+00	8.4E+00	4.5E+04	0.0E+00	0.0E+00
	Std	1.2E+02	1.7E+03	5.4E+01	2.8E+02	0.0E+00	3.8E+01	6.0E+03	0.0E+00	0.0E+00
	Rank	7	8	5	6	2	4	9	2	2
f9	Mean	9.3E+00	9.8E+00	6.1E+00	4.5E+00	0.0E+00	6.0E+00	1.2E+01	1.1E+00	0.0E+00
	Std	3.3E-01	1.8E+00	5.1E+00	7.3E-01	0.0E+00	2.4E+00	3.1E-01	3.1E+00	0.0E+00
	Rank	7	8	6	4	1.5	5	9	3	1.5
f10	Mean	2.6E+00	1.9E+00	2.3E-01	6.2E+00	0.0E+00	8.0E-02	2.4E+01	6.5E-02	0.0E+00
	Std	2.0E-01	1.6E+00	1.2E-01	1.2E+00	0.0E+00	4.1E-02	7.7E-01	5.9E-02	0.0E+00
	Rank	7	6	5	8	1.5	4	9	3	1.5

The functions $f_1$ –$f_{10}$ are unimodal and multimodal functions. These kinds of function are selected after a couple of testing purposes. In particular, unimodal functions allow us to evaluate the exploitation performance of meta-heuristic optimizers since they only have one global optimal minimum; multimodal functions help us see algorithms’ exploration performance with several local minimum points, which exponentially increases following the increase in search space dimension.

In general, it can be seen from Table 2 that ISLO shows the best performance among all algorithms chosen in most test cases except $f_4$ and $f_5$. Furthermore, while optimizing several functions, ISLO can reach optimal value with decent stability.

**Fig. 5:**
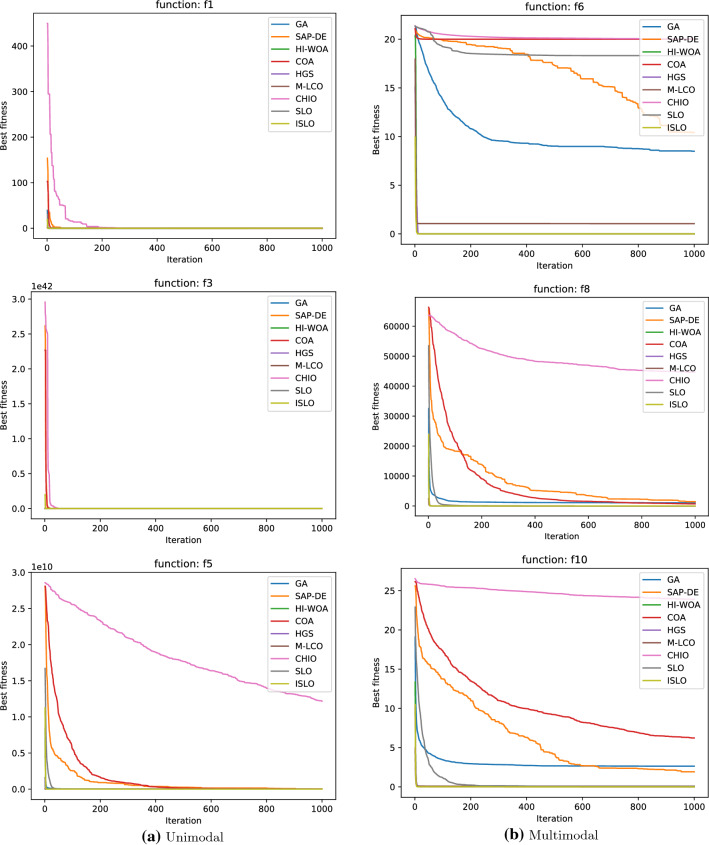
Convergence speed of each algorithm on unimodal (**a**-left side) and multimodal (**b**-right side) functions

***Accuracy and the stability***

**Fig. 6:**
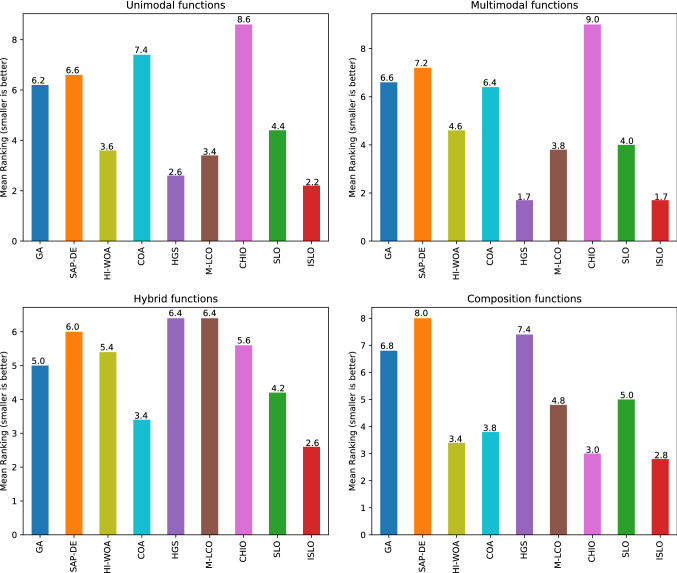
Visualization of mean ranking on unimodal, multimodal, hybrid, and composition functions of compared algorithms

From the obtained results of unimodal and multimodal functions in Table 2, it could be made the following observations:

- ISLO achieves the best results in all test cases except $f_4$ and $f_5$. For example, in experiments with unimodal function $f_1$-$f_5$, ISLO can reach the global optimal, as well as SLO, M-LCO, and HI-WOA models in the *f*1 function. ISLO, M-LCO, and HGS find the global optimal for the functions *f*2 and *f*3. ISLO is ranked 2*nd* in *f*4, *f*5, and the difference between the results of the ranked 1*st* (HI-WOA) and ISLO is not significant. In particular, our improvement makes ISLO outperforms SLO in all test cases. It proves that compared to the original SLO, ISLO’s exploitation ability is significantly enhanced. The results with unimodal functions indicate that ISLO could lead the population to the global optimal position. Furthermore, in addition to the best results in terms of accuracy, ISLO also shows extreme stabilization since the standard deviation values are 0 in all three cases.
- The results for multimodal functions $f_6$–$f_{10}$ indicate that ISLO also has superior exploration ability. ISLO ranks 1*st* in 5 of 5 functions, and with four functions $f_7-f_10$, ISLO reaches the globally optimal values of the functions, accompanied by relatively small standard deviation values. Notably, ISLO’s results outperform SLO’s results in both terms of accuracy and stability (except function *f*7, SLO attains the same 1*st* ranking as ISLO). It is a proof that the exploration ability is significantly improved.
- We also visualize the mean ranking of all algorithms in unimodal and multimodal functions in Fig. 6. ISLO is ranked as the best optimizer for both types of benchmark functions (2.2 for unimodal functions and 1.7 for multimodal functions).

***Convergence characteristic***

The convergence curves in Fig. 5 show that with unimodal and multimodal functions, most algorithms can converge at the exact or near the global optimal point. Especially with unimodal functions such as $f_1$ and $f_3$. Our proposed model ISLO starts to converge very fast right after a few iterations. As can be seen, the results from ISLO are far better than the original SLO in all cases, proving that exploitation and exploration capacities in SLO are considerably enhanced.

#### Hybrid and Composition Functions Results

**Table 3:** Comparison of the optimization results obtained for hybrid functions (f11–f15) and composition functions (f16–f20)

Function		GA	SAP-DE	HI-WOA	COA	HGS	M-LCO	CHIO	SLO	ISLO
f11	Mean	7.88E+06	3.28E+07	4.78E+06	1.39E+07	1.22E+07	1.18E+07	9.22E+06	3.53E+06	2.16E+06
	Std	2.91E+06	2.18E+07	2.53E+06	1.41E+07	9.90E+06	1.06E+07	3.79E+06	1.98E+06	1.58E+06
	Rank	4	9	3	8	7	6	5	2	1
f12	Mean	2.43E+08	3.09E+09	4.22E+07	6.65E+06	1.10E+08	4.34E+08	2.24E+08	4.95E+06	5.61E+05
	std	8.04E+07	1.79E+09	1.03E+08	1.66E+07	1.74E+08	2.78E+08	2.18E+08	2.18E+07	1.53E+06
	Rank	7	9	4	3	5	8	6	2	1
f13	Mean	6.39E+12	1.30E+05	1.83E+14	1.25E+07	8.28E+11	1.32E+12	5.23E+12	7.55E+08	1.62E+05
	Std	3.71E+12	3.59E+04	2.87E+14	5.23E+07	1.58E+12	2.02E+12	1.18E+13	2.85E+09	7.15E+04
	Rank	8	1	9	3	5	6	7	4	2
f14	Mean	6.293E+02	6.431E+02	6.405E+02	6.258E+02	6.418E+02	6.401E+02	6.228E+02	6.353E+02	6.341E+02
	Std	1.214E+00	1.826E+00	2.064E+00	3.218E+00	1.896E+00	2.601E+00	1.262E+00	2.750E+00	2.892E+00
	Rank	3	9	7	2	8	6	1	5	4
f15	Mean	1.028E+03	1.018E+03	1.089E+03	9.867E+02	1.143E+03	1.127E+03	1.276E+03	1.227E+03	1.106E+03
	Std	1.395E+01	4.438E+01	7.539E+01	4.116E+01	3.242E+01	3.788E+01	4.634E+01	3.156E+01	3.189E+01
	Rank	3	2	4	1	7	6	9	8	5
f16	Mean	9.132E+02	9.136E+02	9.134E+02	9.136E+02	9.136E+02	9.133E+02	9.132E+02	9.130E+02	9.129E+02
	Std	1.328E-01	2.035E-01	3.438E-01	2.409E-01	2.430E-01	3.812E-01	2.358E-01	3.391E-01	3.592E-01
	Rank	4	7	6	9	8	5	3	2	1
f17	Mean	3.99E+07	8.11E+07	3.76E+06	6.52E+06	5.87E+07	5.35E+07	6.55E+06	3.16E+07	2.80E+07
	Std	4.57E+06	3.05E+07	3.25E+06	5.13E+06	2.49E+07	3.49E+07	2.86E+06	1.43E+07	1.24E+07
	Rank	6	9	1	2	8	7	3	5	4
f18	Mean	3.29E+11	7.81E+13	9.19E+03	2.69E+09	1.21E+13	1.46E+11	1.10E+04	1.04E+10	1.11E+04
	Std	4.73E+11	1.76E+14	3.55E+03	1.20E+10	4.41E+13	2.80E+11	3.74E+03	3.42E+10	3.81E+03
	Rank	7	9	1	4	8	6	2	5	3
f19	Mean	9.06E+03	7.06E+03	4.74E+03	2.11E+03	4.30E+03	2.87E+03	4.82E+03	6.87E+03	1.73E+03
	Std	3.51E+02	2.48E+03	1.52E+03	1.26E+02	1.16E+03	6.53E+02	1.20E+03	1.60E+03	3.78E+01
	Rank	9	8	5	2	4	3	6	7	1
f20	Mean	3.018E+03	2.951E+03	2.829E+03	2.745E+03	3.044E+03	2.800E+03	2.331E+03	2.911E+03	2.839E+03
	Std	3.659E+01	7.323E+01	6.992E+01	2.421E+02	4.993E+01	5.779E+01	1.579E+02	4.414E+01	6.245E+01
	Rank	8	7	4	2	9	3	1	6	5

The functions $f_{11}$–$f_{20}$ are hybrid and composition functions. In hybrid functions ($f_{11}$–$f_{15}$, the variables are randomly divided into sub-components which play a role as input for different basic functions including both unimodal and multimodal functions. To work well on these functions, algorithms are required an extreme balance between exploitation and exploration phases, because hybrid functions are both unimodal and multimodal, and they own different properties for different variables sub-components. On the other hand, optimization of composite mathematical functions ($f_{16}$–$f_{20}$) is a very challenging task, because local optima are only avoided by a proper balance between exploitation and exploration.

**Fig. 7:**
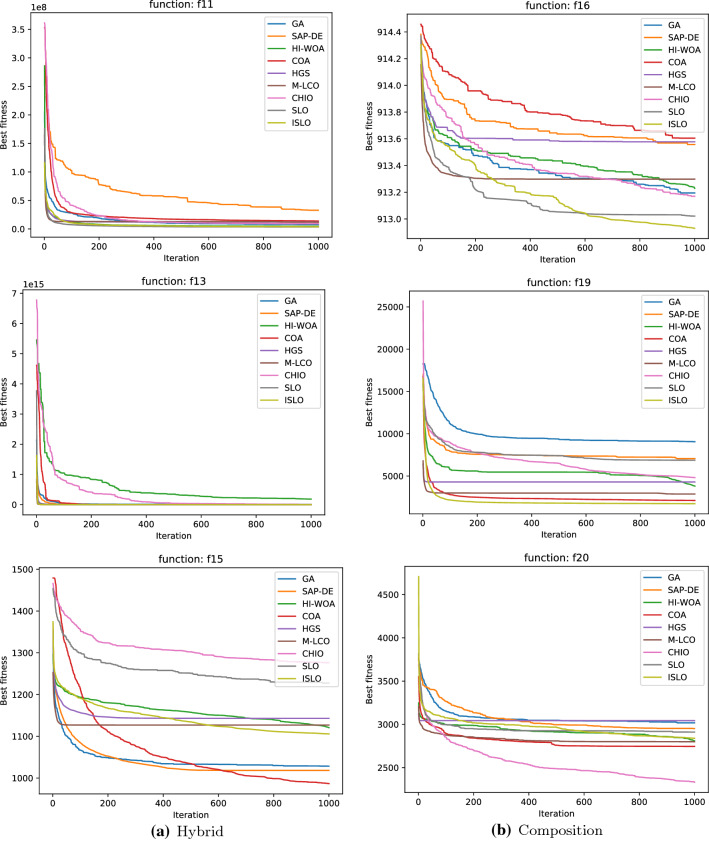
Convergence speed of each algorithm on hybrid (**a**-left side) and composition (**b**-right side) functions

In general, Table 3 shows that ISLO achieves competitive results performance overall hybrid and composition functions. ISLO results rank first in several cases such as $f_{11}, f_{12}, f_{16}$ and $f_{19}$. Also, as is observed in Fig. 7, ISLO’s convergence curves are similar to those in unimodal and multimodal functions, and ISLO still has a very fast convergence after the first half of iteration because of its updating mechanism.

***The accuracy and the stability***

The following comments are drawn from Table 3.

- Evidently that ISLO works well for hybrid functions ($f_{11}$–$f_{15}$). In particular, it shows superior results for functions $f_{11}$ and $f_{12}$ compared to state-of-the-art algorithms such as HI-WOA and HGS. In the case of the function $f_{13}$, although ISLO does not account for the first place, it is still very competitive when its result is only worse than SAP-DE. Even in the case of functions $f_{14}$ and $f_{15}$, ISLO’s results are still better than SLO’s results, proving a decent balance between the exploitation and exploration phases, especially when compared with the original SLO algorithm.
- For composition functions ($f_{16}$–$f_{20}$), ISLO performance presents competitive results to other models. ISLO ranked 1*st* place when solving functions $f1_{16}$ and $f_{19}$. Specifically, in function $f_{18}$ there is no big differences between ISLO’results and the best one HI-WOA and the second best CHIO. Furthermore, the results of ISLO again outperform the results of SLO in all cases. It proved that our improvement makes ISLO better than traditional one.
- ISLO ranking (Fig. 6) is not at the 1*st* place in most cases (3/5). But overall ranking (mean ranking) shows that ISLO is the best optimizer on both benchmark function types (2.6 in hybrid functions and 2.8 in composition functions).

***The convergence speed***

The convergence speed of all algorithms that work on hybrid and composition functions is shown in Fig. 7. Since these functions are difficult to converge on, only powerful and efficient algorithms can converge to the optimal point. Therefore, we can observe which algorithm works and which algorithm does not work in these figures.

It is clear that ISLO always considerably improves its best global fitness values in the second half of the iterations. The reason is that in the first half of the iterations, ISLO is in its exploration phase (since the value of *C* during that time is always greater than 1, see Algorithm 2). After changing to the exploitation phase, ISLO can exploit and converge to the global minimum quickly, providing better results than the others.

### Practical Test Results

**Table 4:** Comparison among models on each dataset by different measurements

Dataset	Model	MAE	RMSE	MAPE	KGE	KLD
Google Trace CPU	MLP	**0.075**	**0.099**	**0.089**	0.843	2.525
	CFNN	0.080	0.103	0.095	0.847	**2.311**
	FLNN	0.132	0.159	0.170	0.700	3.693
	ELM	0.090	0.119	0.111	0.753	2.508
	OTWO-ELM	0.127	0.166	0.158	**0.585**	2.360
	SLO-ELM	0.113	0.150	0.138	0.662	2.552
	ISLO-ELM	0.080	0.105	0.098	0.755	2.361
Google Trace RAM	MLP	0.014	0.016	0.070	0.866	2.804
	CFNN	**0.008**	**0.010**	**0.039**	0.886	1.876
	FLNN	0.010	0.013	0.048	0.906	1.809
	ELM	0.009	0.013	0.045	**0.834**	1.897
	OTWO-ELM	0.011	0.015	0.052	0.850	1.835
	SLO-ELM	0.009	0.012	0.043	0.883	1.622
	ISLO-ELM	0.009	0.013	0.041	0.883	**1.599**
EU Internet Traffic	MLP	4.793	7.680	0.014	0.994	4.698
	CFNN	4.848	**7.340**	0.014	0.993	4.865
	FLNN	5.381	7.649	0.016	**0.987**	4.617
	ELM	6.135	9.208	0.017	0.993	**4.506**
	OTWO-ELM	6.434	9.470	0.018	0.995	4.593
	SLO-ELM	5.295	7.859	0.014	0.990	4.515
	ISLO-ELM	**4.751**	**7.340**	**0.013**	0.995	4.703
UK Internet Traffic	MLP	4.635	8.234	**0.009**	0.995	4.817
	CFNN	4.777	8.321	**0.009**	0.996	4.899
	FLNN	4.971	8.648	0.010	0.994	4.996
	ELM	4.754	8.589	0.010	**0.993**	4.918
	OTWO-ELM	4.852	8.543	0.010	0.988	4.902
	SLO-ELM	4.810	8.441	0.010	**0.993**	**4.755**
	ISLO-ELM	**4.549**	**8.142**	**0.009**	**0.993**	4.774

The best value of each metric on each dataset is highlighted (as bold values)

**Fig. 8:**
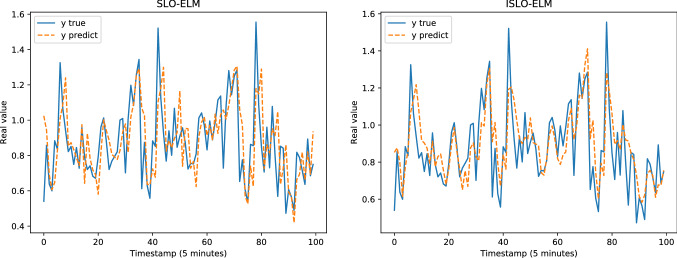
Prediction results of SLO-ELM and our ISLO-ELM on Google trace CPU data

**Fig. 9:**
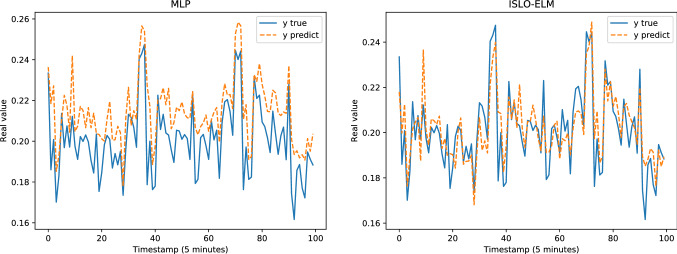
Prediction results of MLP and our ISLO-ELM model on Google trace RAM data

**Fig. 10:**
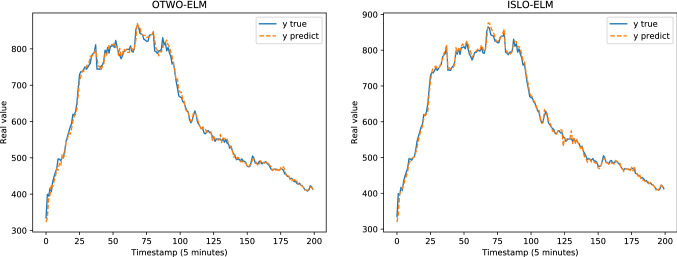
Prediction results of OTWO-ELM and our ISLO-ELM model on the UK Internet traffic data

**Fig. 11:**
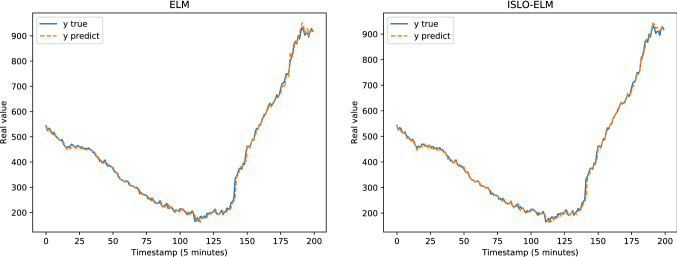
Prediction results of ELM and our ISLO-ELM model on the EU Internet traffic data

Table 4 presents the results of all models in each dataset evaluated by MAE, RMSE, MAPE, KGE and KLD measurements. Figure 8 illustrates the comparison between the predicted output and the ground truth of the models for the Google trace CPU dataset. Figure 9 shows the performance of different optimizers, including OTWO, SLO, ISLO and the original ELM of the Google trace RAM dataset (Figs. 10 and 11). In general, our proposed ISLO-ELM model is very competitive in working on all datasets with most metrics such as MAE, RMSE, MAPE, and KLD metrics metrics. Especially, it works well for the Internet traffic EU and UK datasets.

- For the Google trace CPU dataset, MLP shows the best results with MAE, RMSE and MAPE (3/5 metrics). Of the hybrid models, only OTWO-ELM shows the best result with KGE. Although ISLO-ELM belongs to the better ones, its improvements for SLO are significant.
- For the Google trace RAM dataset, CFNN shows the best performance when it reaches the best results on 3/5 metrics MAE, RMSE and MAPE. ISLO-ELM achieves the best results only on the KLD. The improved operators used in the ISLO algorithm are also efficient compared to the traditional SLO for this data set.
- For the EU Internet traffic dataset, ISLO-ELM shows the best results with MAE, RMSE, MAPE (3/5 metrics).
- For the UK Internet traffic dataset, ISLO-ELM shows the best results with MAE, RMSE, MAPE and KGE (4/5 metrics).

## Conclusion and Future Works

In this paper, we proposed an improved version of Sea Lion Optimization which is based on the historical movement of sea lions in combination with the levy flight trajectory and the idea of the opposition-based learning method, forming an Improved Sea Lion Optimization (ISLO). The effectiveness of ISLO is proved by both theoretical and practical experiments.

In theoretical tests, 20 benchmark functions are used, and the ISLO’s results are compared with eight recent metaheuristic algorithms. In practical experiments, four different real and public datasets are used. The results of the proposed ISLO-ELM are better than traditional models and hybrid models in most cases, especially with Internet traffic datasets. The results in both tests show that the proposed ISLO outperforms the original SLO algorithm in balancing the exploration and exploitation phase, also in finding global optima. The hybrid ISLO-ELM is also better than several traditional and hybrid models in optimizing neural networks with real-world applications.

In the future, the auto-scaling module is planned to be used in decentralized systems. The autoscaling system includes two main components: the forecasting module and the decision-making module. The forecasting module will use designed models such as ISLO-ELM to predict the incoming values. Meanwhile, the decision-making module takes the information from the forecasting module and gives the final decision on the resource. In such systems, data curation is also very important to monitor covariate shift, which can degrade model performance.

## Data Availability

The authors declare that the data used in this article are available to the public as presented in Sect. 4.2.1.

## Abbreviations

ABC: Artificial bee colony
ADF: Augmented Dickey-Fuller
ARIMA: Autoregressive integratedmoving average
ARMA: Autoregressive moving average
CFNN: Cascade forward neural network
CHIO: Coronavirus herd immunity optimization
COA: Coyote optimization algorithm
CPU: Central processing unit
DE: Differential evolution
ELM: Extreme learning machine
FLNN: Functional-linked neural network
GA: Genetic algorithm
HGS: Hunger game search
$HI-WOA$: Hybrid improved WOA
IaaS: Infrastructure-as-a-Service
ISLO: Improved sea lion optimization
KGE: Kling–Gupta efficiency
KLD: Kullback–Leibler divergence
LCBO: Life choice-based optimization
LFT: Levy fight trajector
MA: Moving average
MAE: Mean absolute error
MAPE: Mean absolute percentage error
MHM: Memorizing historical movement
$M-LCO$: Modified version of LCBO
MLP: Multi-layer perceptron
MSE: Mean squared error
NN: Neural network
OBL: Opposition-based learning
OTWO: Enhanced tug of war optimization
PSO: Particle swarm optimization
QSO: Qeuing search optimization
RAM: Random access memory
RMSE: Root mean squared error
$SAP-DE$: Surrogate assisted parameter adapted DE
SLO: Sea lion optimization
STL: Seasonal-trend decomposition using locally estimated scatterplot smoothing
WOA: Whale optimization algorithm
